# Evaluation of the Antioxidant Capacity of Synthesized Coumarins

**DOI:** 10.3390/ijms13067260

**Published:** 2012-06-13

**Authors:** Damiana R. Vianna, Guilherme Bubols, Gabriela Meirelles, Bárbara V. Silva, Alessandra da Rocha, Maurício Lanznaster, José Maria Monserrat, Solange Cristina Garcia, Gilsane von Poser, Vera Lucia Eifler-Lima

**Affiliations:** 1 Post-Graduate Program in Pharmaceutical Sciences, Federal University of Rio Grande do Sul, Av. Ipiranga, 2752, 90610-000, Porto Alegre, RS, Brazil; E-Mails: 00121940@ufrgs.br (D.R.V.); bubols@hotmail.com (G.B.); gabimeirelles@gmail.com (G.M.); gilsane.von@ufrgs.br (G.P.); 2 Institute of Chemistry, Federal University of Rio de Janeiro, UFRJ, Bloco A, Cidade Universitária, 21941-909, Rio de Janeiro, RJ, Brazil; E-Mail: barbara.iq@gmail.com; 3 Post-Graduate Program in Physiological Sciences, Comparative Animal Physiology, Institute of Biological Sciences, Federal University of Rio Grande, FURG, Av Itália km 8 s/n, 96201-900, Rio Grande, RS, Brazil; E-Mails: alessandramr@gmail.com (A.R.); josemmonserrat@gmail.com (J.M.M.); 4 Institute of Chemistry, Universidade Federal Fluminense, UFF, Outeiro de São João Batista, s/n, Campus do Valonguinho Centro, 24020-150, Niterói, RJ, Brazil; E-Mail: mlanz@vm.uff.br

**Keywords:** antioxidant capacity, peroxyl radicals, coumarins, DPPH, cyclic voltammetry

## Abstract

Coumarins are secondary metabolites that are widely distributed within the plant kingdom, some of which have been extensively studied for their antioxidant properties. The antioxidant activity of coumarins assayed in the present study was measured by different methods, namely the 1,1-diphenyl-2-picryl-hydrazyl (DPPH^•^) method, cyclic voltammetry and the antioxidant capacity against peroxyl radicals (ACAP) method. The 7,8-dihydroxy-4-methylcoumarin (LaSOM 78), 5-carboxy-7,8-dihydroxy-4-methylcoumarin (LaSOM 79), and 6,7-dihydroxycoumarin (Esculetin) compounds proved to be the most active, showing the highest capacity to deplete the DPPH radicals, the highest antioxidant capacity against peroxyl radicals, and the lowest values of potential oxidation.

## 1. Introduction

Coumarins are naturally occurring compounds, widely distributed throughout the plant kingdom. Some of them are physiologically active and have recently been extensively studied for their antioxidant properties. In fact, several studies have shown that coumarins elicit antitumoral [1,2], anti-inflammatory [3,4] and neuroprotective effects [5,6], and that the inhibition of oxidative stress is a contributing factor in each of these biological effects. Indeed, coumarins have been reported to affect the formation and scavenging of reactive oxygen species (ROS) and influence free radical-mediated oxidative damage, thus the development of new synthesized coumarin molecules is promising, especially regarding their antioxidant activities. In this context, the antioxidant capacity of coumarins with different chemical substitutions has been previously investigated by free radical-scavenging assays, including 1,1-diphenyl-2-picrylhydrazyl (DPPH^•^) radical [7], in order to evaluate their antioxidant behavior.

In addition to chemical assays such as the DPPH^•^ radical, a proper determination of the antioxidant potential of new compounds is usually carried out by confirmation with further techniques. In this scenario, voltammetric methods represent an attractive analytical option for the rapid screening of large quantities of compounds in the search for novel antioxidants. However, cyclic voltammetry in comparison to several established techniques has not found widespread use, despite the fact that some studies have recommended the use of this technique as an instrumental tool in evaluating the total antioxidant capacity of natural products [8–11]. Nevertheless, it remains necessary to utilize cyclic voltammetry along with well-established techniques in order to understand the antioxidant behavior of synthetic compounds. Moreover, fluorometry has also been used in the analysis of potential antioxidant compounds, as represented by the antioxidant capacity against peroxyl radicals (ACAP), which is another relevant method that has been described to evaluate the antioxidant competence of samples against peroxyl radicals [12].

The adoption of chemical, electrochemical and fluorometric methods provides a thorough approach in the investigation of antioxidant properties of coumarins and allows a comparison among them, especially in the optimization of unconventional techniques such as cyclic voltammetry. Therefore, this report sought to investigate the radical scavenging capacities using the DPPH^•^ spectrophotometric assay, the cyclic voltammetric (CV) characterization, and the antioxidant capacity against peroxyl radicals (ACAP) of nine coumarins obtained from synthesis and also commercially available coumarins.

## 2. Results and Discussion

Several recent studies have shown the ability of coumarins to reduce ROS production and consequently the damage to lipids, protein and DNA found in various biological processes, for instance in cancer [13,14] and inflammatory conditions [4,15]. In light of these studies, there is clearly a current interest in developing molecules with high antioxidant activity as potential drug candidates to be adopted against these pathologies, and coumarins stand out as promising antioxidants with pharmacological interest [16,17]. In our study, one of the assays employed was the evaluation of antioxidant activity toward the radical DPPH^•^. Prior to the quantitative studies of DPPH^•^ bleaching by coumarins, a preliminary test was carried out using the DPPH-TLC method. When the plates with coumarins were sprayed with a DPPH^•^ solution (0.2%), 7,8-dihydroxy-4-methylcoumarin (LaSOM 78), 5-carboxy-7,8-dihydroxy-4-methylcoumarin (LaSOM 79), and 6,7-dihydroxycoumarin (Esculetin) showed a pronounced DPPH^•^ bleaching capacity.

**Figure 1:**
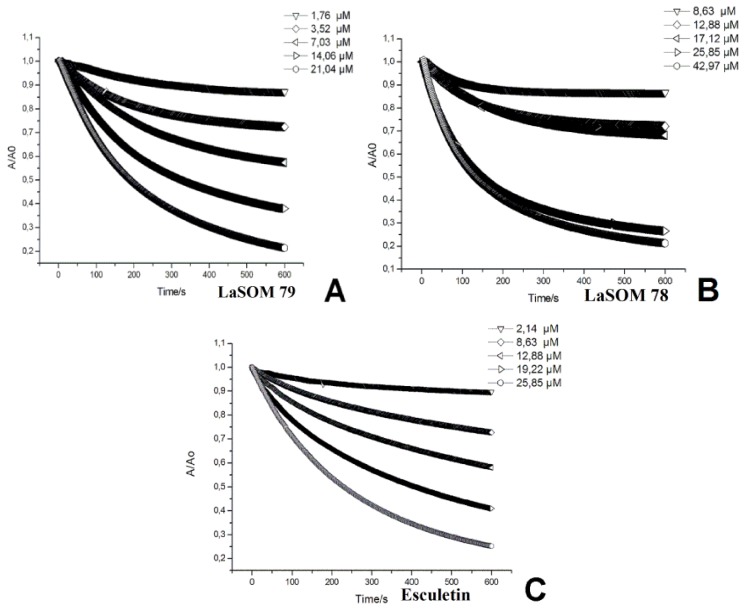
Kinetics profiles of DPPH^•^ bleaching by coumarins: LaSOM 79 (**A**), LaSOM 78 (**B**) and Esculetin (**C**).

Considering these results, the antioxidant activity of these three coumarins was quantitatively evaluated following the DPPH^•^ absorbance. When the coumarins (1.76 to 42.97 μM) were added to a DPPH^•^ solution (60 μM), a fast bleaching of DPPH^•^ was observed (Figure 1). The kinetic profile shows that these coumarins were able to bleach DPPH^•^ in a concentration-dependent manner, in which the 5-carboxy-7,8-dihydroxy-4-methylcoumarin represented the coumarin with the highest antioxidant capacity against this radical.

**Figure 2:**
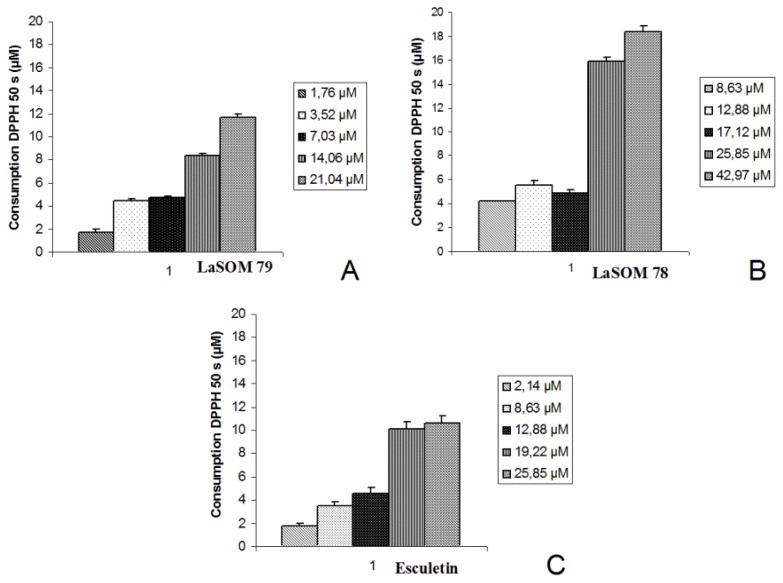
Comparison among the DPPH^•^ consumption (consumption of 50 s) of LaSOM 79 (**A**), LaSOM 78 (**B**) and Esculetin (**C**). The results are expressed as mean ± standard deviation.

The kinetic profiles of DPPH^•^ bleaching induced by the coumarins showed a biphasic decay. A fast absorbance decrease is observed in the first seconds, which over longer reaction times, then followed by a considerably slower process (Figure 2). These profiles imply the presence of two or more reactive centers of different reactivity [18]. This multiplicity of reactive centers can be taken into account by fitting the decay to a bi-exponential function (Equation (1)). The percentage of DPPH^•^ consumption was calculated according to Equation 2.

In general, these results demonstrate that Esculetin shows a higher radical consumption in the first 50 s of reaction, indicating that this sample has high reactivity with the DPPH^•^ radical. Although 5-carboxy-7,8-dihydroxy-4-methylcoumarin presents the higher capacity of bleaching the DPPH^•^ radicals, Esculetin seems to be the most reactive among the tested compounds.

IC_50_ values were obtained by linear regression and showed a good coefficient of determination (*r*^2^ ≥ 0.9375). Table 1 shows the IC_50_ values of the tested coumarins when compared to Quercetin.

**Figure 3:**
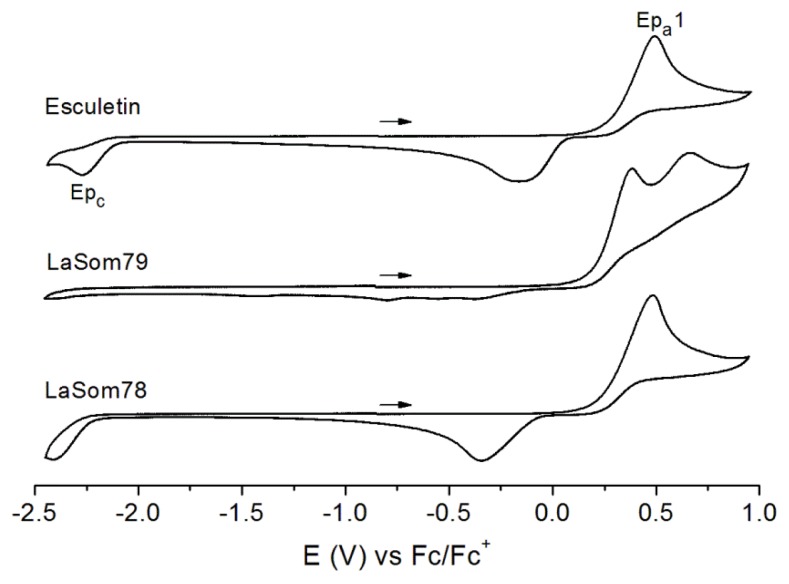
Cyclic voltammograms of Esculetin, LaSOM 79 and LaSOM 78.

A study of the electrochemical properties of the coumarins was performed by cyclic voltammetry, in which an Esculetin-like redox response was observed for the active compounds LaSOM 79 and LaSOM 78 (Figure 3). Except for Quercetin and LaSOM79, which present a second irreversible oxidation peak at 0.45 and 0.67 V *vs.* Fc/Fc^+^, respectively, one irreversible oxidation is observed in the range of 0.22 to 0.49 V *vs.* Fc/Fc^+^ for the active coumarins (Quercetin, Esculetin, LaSOM 78 and LaSOM 79), and in the range of 0.70 to 0.85 V *vs.* Fc/Fc^+^ for the inactive ones (LaSOM 77, LaSOM 80, LaSOM 83, LaSOM 86 and Unbelliferone). The peak potentials and IC_50_ values from the antioxidant activity evaluation are summarized in Table 1. According with these data, the radical scavenging capacity of Quercetin, Esculetin, LaSOM 78 and LaSOM 79 seems to be related with their low oxidation potentials, which are on average 0.36 V lower than those of the inactive coumarins. This significant difference in *E_pa_* values between the two groups of compounds may be associated with the distinct structural features. The highest *E_pa_* value was found for 7-hydroxy-4-methylcoumarin (LaSOM 77) and Umbelliferone, which have a single hydroxyl group in the benzyl ring. Moreover, the coumarins that present no free hydroxyl groups in the ring (LaSOM 83, LaSOM 80, LaSOM 86) also present high *E_pa_* values. In contrast, the presence of two hydroxyl groups on the benzyl ring (as in Esculetin, LaSOM 78, and LaSOM 79) makes the coumarins easier to oxidize; therefore, the value of *E_pa_* is reduced. Overall, the preliminary results indicate that coumarins with *E_pa_* values of up to 0.5 V *vs.* Fc/Fc^+^ present antioxidant activity, while those with higher oxidation potentials proved to be inactive.

**Figure 4:**
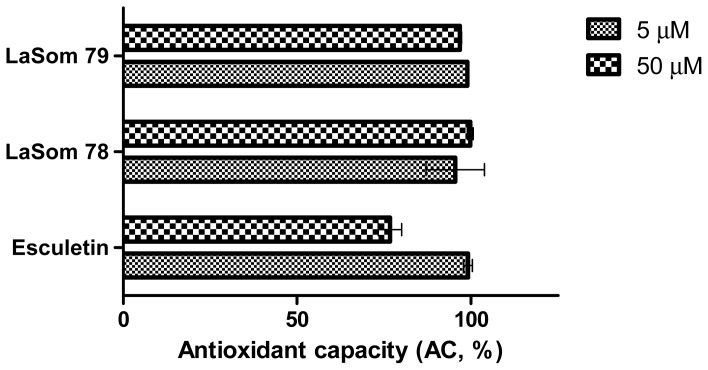
Antioxidant capacity against peroxyl radicals of the tested coumarins LaSOM 79, LaSOM 78, and Esculetin as standard compound. Results are representative of three different experiments and mean ± SD are expressed.

In addition to the previous tests, further analyses were carried out to determine the antioxidant capacity against peroxyl radicals, which provides more specific information concerning the type of radicals scavenged by the coumarins. Figure 4 shows the results from the fluorometric analysis of the tested compounds. Note that, with this methodology, it was possible to confirm the high antioxidant capacity of the synthesized molecules of LaSOM 78, LaSOM 79, and Esculetin. In this manner, the data obtained in our studies show a profile regarding the antioxidant properties of coumarins, referring not only to low reduction potential radicals such as DPPH^•^ (+ 0.25 V) [19], but also to peroxyl radicals, which present a much higher reduction potential (+ 0.71 to + 1.44 V) [20].

Moreover, these results indicate the capacity of the coumarin molecules to scavenge peroxyl radicals. We provide not only information about the extent of antioxidant capacity elicited by the coumarins, but also indicate that the peroxyl radicals represent a kind of reactive species against which the coumarins are able to act. Also, our findings are in agreement with a previous study [19] that indicated a good antioxidant activity of 4-methylcoumarins against peroxyl radicals produced from 2,2′-azobis(2-amidinopropane)-hydrochloride (AAPH), which undergoes a similar thermal decomposition to the ABAP used in the present study.

Taken together, our results indicate that the activity of our synthesized coumarins are correlated with the number of hydroxyl groups, which in turn indicated that the chemical structure and the number of hydroxyl groups within the moiety group are directly correlated with the effects of ROS suppression. The hydroxyl group showed a great influence, while the methyl group presented the lowest influence. In addition, it could be observed that the presence of the carboxyl group strongly improves the antioxidant activity.

## 3. Experimental Section

### 3.1. Test Compounds

**Figure 5:**
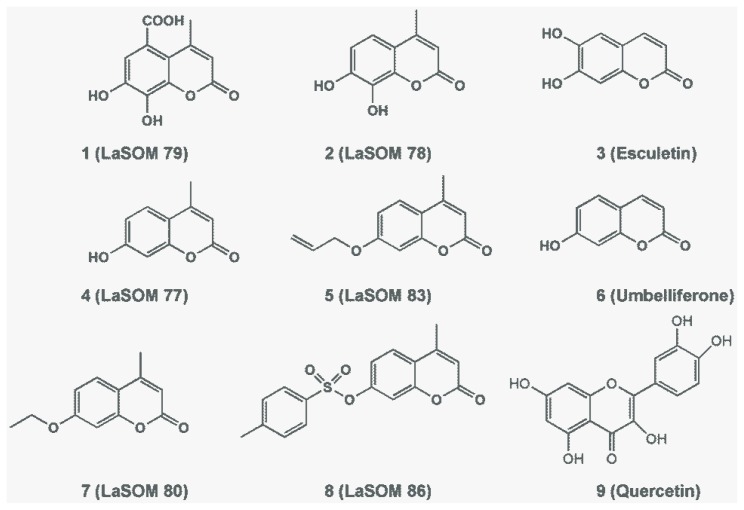
Structures of the tested coumarins. LaSOM 79 (**1**), LaSOM 78 (**2**), Esculetin (**3**), LaSOM 77 (**4**), LaSOM 83 (**5**), Umbelliferone (**6**), LaSOM 80 (**7**), LaSOM 86 (**8**) and Quercetin (**9**).

The tested coumarins (Figure 5) were synthesized by the well-known Pechmann condensation technique, as described in prior literature [21]. The Microwave Pechmann reaction of resorcinol with ethyl acetoacetate in the presence of catalytic amounts of concentrated HCl led to 7-hydroxy-4-methyl-2*H*-chromen-2-one (LaSOM 77). The same conditions led to the formation of LaSOM 78 (7,8-dihydroxy-4-methylcoumarin) and LaSOM 79 (5-carboxy-7,8-dihydroxy-4- methylcoumarin) from pyrogallol and gallic acid, respectively. In order to explore the influence of the substitution at the 7- position of coumarins in the antioxidant activity, LaSOM 77 was submitted to reactions with a variety of electrophiles to give 7-*O*-substituted coumarins (unpublished data). Commercially available coumarins, Umbelliferone and Esculetin, were also tested. Quercetin was used as a positive control and was prepared in the same concentration range as the tested coumarins.

### 3.2. DPPH Bioautographic Assay

The assay known as bioautographic DPPH^•^ was first performed as a screening method based on thin layer chromatography (TLC) bioautography [22]. This technique allows for the evaluation of the antioxidant activity of samples due to their capacity to neutralize the stable free radical DPPH^•^. For this assay, all coumarins and Quercetin were solubilized in an acetone:methanol solution (1:1).

#### DPPH Spectrophotometric Assay

The quantitative evaluation of antioxidant activity against the DPPH^•^ radical was performed by the spectrophotometric measurement of DPPH^•^ consumption in the presence of antioxidants [23]. Briefly, aliquots of test samples in acetone:methanol (1:1, *v*/*v*) in different concentrations were added to an ethanolic DPPH^•^ solution (molar absorption coefficient 517 nm: 11500 M^−1^·cm^−1^). Measurements started immediately after mixing the solutions. The absorbance decrease of DPPH^•^ was evaluated after 50 or 600 s and monitored at *λ* = 517 nm and 25 °C with measurements every 5 s, followed by measurements at 10 s intervals when the reaction is in their plateau state, with a total reaction time of 600 s for all samples. For the evaluation of the antioxidant capacity, experimental data (kinetics profiles of DPPH^•^ decay) were adjusted to a bi-exponential equation (Equation (1)). DPPH^•^ and coumarin solutions were prepared daily. The experiments were performed in triplicate, and the results were expressed as the average of *A*/*A*_o_. IC_50_ values were calculated for each sample and denote the concentration of samples required to scavenge 50% of DPPH^•^ radicals. Ethanol was used as a blank, and Quercetin was employed as a reference compound.

(1) $${\text{Abs}}_{\text{t}}=y_{\text{o}}+A_{1}\;{\text{exp}}^{\left(-t/T1\right)}+A_{2}\;{\text{exp}}^{\left(-t/T2\right)}$$

The activity of the tested compounds was characterized by:

(A_1_ + A_2_) = total bleaching capacity.
(Abs_0_ − Abs_50_) = consumption of DPPH^•^ at a short reaction time (50 s). This takes into account only fast processes.
(Abs_0_ − Abs_600 s_) = consumption of DPPH^•^ over a long reaction time (600 s). This takes into account fast and slow processes.

The percentage of DPPH^•^ consumption was calculated according to Equation (2) and compared among the different samples.

(2) $$\text{Consumption }50\;\text{s}={\text{Abs}}_{0}-{\text{Abs}}_{50}/{{ɛ}_{\text{DPPH}}}^{•}$$

where Abs_0_ is the absorbance at *t* = 0 s, Abs_50_ is the absorbance before 50 s of reaction, and *ɛ*_DPPH_^•^ is the molar absorption coefficient of DPPH^•^.

### 3.3. Cyclic Voltammetry

Cyclic voltammetry experiments were recorded using a BAS Epsilon potentiostat-galvanostat with a regular three component cell: a glassy carbon used as a working electrode, Ag/AgCl reference electrode for organic media, and a platinum wire as a counter electrode. The compounds were dissolved in anhydrous dimethylsulfoxide (DMSO, Sigma-Aldrich) with 0.1 mol·L^−1^ of TBAClO_4_ (Sigma-Aldrich, electrochemical grade), in a concentration of 2 mg·mL^−1^, and purged with ultrapure argon before each measurement. The potential was cycled from −2.5 V to 1.0 V and back to −2.5 V *vs.* Fc/Fc^+^, at a scanning rate of 100 mV/s. Ferrocene was added at the end of each experiment as an internal standard (*E*_1/2_ = 0.40 V *vs.* NHE) [24].

### 3.4. Antioxidant Capacity against Peroxyl Radicals

The assay to evaluate the total antioxidant capacity against peroxyl radicals (ACAP) is based on that described by Amado *et al*. [12], with the following modifications. Peroxyl radicals are generated by thermal decomposition at 37 °C of 2,2′-azobis(2-methylpropionamidine) dihydrochloride (ABAP, Sigma-Aldrich) in the analyzed samples, resulting in the emission of a fluorescent signal caused by the reaction between ROS and 2′,7′-diclorodihidrofluorescein (H_2_DCF), which had previously been deacetylated in an alkaline solution (see below).

Briefly, 10 μL of the diluted samples at 5 and 50 μM were pipetted into a white 96-well microplate, six wells per sample. The blanks were prepared by transferring the same volume of solvent to the microplate for each of the solvents necessary to dissolve the samples, according to their solubility. Esculetin, LaSOM 78, and LaSOM 79 were solubilized in ethanol, while all the other coumarins were solubilized in DMSO. Blanks were prepared with and without ABAP, as well as with and without H_2_DCF. The reaction buffer (127.5 μL), containing 30 mM HEPES (pH 7.2), 200 mM KCl, and 1 mM MgCl_2_, was added to the samples. After, 7.5 μL of 10 mM ABAP was added to three wells of each sample, while the same volume of ultrapure water (Milli-Q) was added to the remaining wells.

Immediately before the microplate reading, 10 μL of H_2_DCF was added to the wells at a final concentration of 40 μM. 2′,7′-dichlorodihydrofluorescein diacetate (H_2_DCF-DA, Invitrogen) was previously cleaved by alkaline hydrolysis for 30 min, resulting in the deacetylated product H_2_DCF. The microplates were then put into Victor2 D, Perkin Elmer fluorescence microplate reader (Waltham, MA, USA) programmed to maintain the temperature at 37 °C in order to induce ABAP thermolysis, thus producing peroxyl radicals. Next, the H_2_DCF nonfluorescent compound is oxidized by ROS into the DCF fluorescent compound, which is detected at 485 and 520 nm, as the excitation and emission wavelengths, respectively, for 90 min, with readings every 5 min.

Total fluorescence production was calculated according to Equation 3, and the results were expressed in percentage (w/ and w/o meaning with and without, respectively).

(3) ACAP(%)=(ΔBlank-ΔSample)/ΔBlank×100

in which ΔBlank = NF Blank w/ ABAP − NF Blank w/o ABAP; ΔSample = NF Sample w/ ABAP − NF Sample w/o ABAP; NF (Net Fluorescence) = AF w/ H2DCF − AF w/o H2DCF; AF = Average Fluorescence, calculated from each triplicate.

### 3.5. Statistical Analysis

The experiments were performed in triplicate. DPPH results are given as *A*/*A*_o_. One-way analysis of variance (ANOVA) was used to compare more than two means. A difference was considered statistically significant when *p* ≤ 0.05.

## 4. Conclusions

As a result of the different techniques employed in this study, we were able to demonstrate that 5-carboxy-7,8-dihydroxy-4-methylcoumarin (LaSOM 79), 7,8-dihydroxy-4-methylcoumarin (LaSOM 78), and 6,7-dihydroxycoumarin (Esculetin), whose structures present two hydroxyl moieties on the benzene rings, proved to be the three most effective antioxidant coumarins in all the techniques employed. These active compounds exhibited a low potential range, due to an increase in electron density on the aromatic ring, which provided a reduction of DPPH^•^ and peroxyl radicals.

## Figures and Tables

**Table 1:** Radical scavenging potential and anodic potentials of tested compounds. IC_50_: half maximal inhibitory concentration, obtained from DPPH assay; *E_pa_*: anodic potential, obtained from cyclic voltammetry *vs.* Fc/Fc^+^.

Compounds	IC_50_ (μM)	*E_pa 1_* (V)	*E_pa 2_* (V)
LaSOM 79	17.49	0.44	0.67
LaSOM 78	33.46	0.49	-
Esculetin	25.18	0.48	-
Quercetin	29.41	0.22	0.45
LaSOM 77	-	0.85	-
LaSOM 83		0.76	-
Umbelliferone	-	0.83	-
LaSOM 80	-	0.72	-
LaSOM 86	-	0.70	-
